# ‘Midlife care to migrant women: primary healthcare providers’ beliefs about barriers and facilitators’

**DOI:** 10.1017/S1463423625000349

**Published:** 2025-05-08

**Authors:** Karin Stanzel, Mary Pham, Karin Hammarberg, Jane Fisher

**Affiliations:** 1 Global and Women’s Health, School of Public Health and Preventive Medicine, Monash University, Melbourne, Australia; 2 Faculty of Medicine, Nursing and Health Sciences, Monash University, Clayton, Australia

**Keywords:** Menopause, midlife, migration experiences, migrant women, Primary healthcare, primary healthcare providers

## Abstract

**Aim:**

This study aimed to understand primary healthcare providers’ beliefs about barriers and facilitators providing culturally competent midlife care to migrant women.

**Background:**

Primary healthcare is the entry level to the health system. It is usually the first point of contact in accessing the healthcare system and provides a range of services including health promotion and prevention. Migrant women are less likely to access and engage in health screening and health promotion activities and consequently may miss out on optimal health in older age.

**Methods:**

A cross-sectional study including two free-text questions, part of an online survey, was thematically analysed. 76 primary healthcare providers answered the free-text questions.

**Findings:**

Competing priorities as a result of migration and settlement experiences, the healthcare systems’ limited resources to respond to the needs of migrant population and culturally informed beliefs and behaviour about menopause were viewed as barriers to midlife care for migrant women. Flexible models of primary healthcare and coordinated engagement with community groups were proposed to address these barriers. Primary healthcare providers perceived the current primary healthcare model to be inadequate to address the additional needs of migrant women. A review of the model of care may include ‘task shifting’ where nurses provide advanced care to migrant women in midlife. Perceptions of midlife and menopause are informed by culture. Hence, a culturally informed health promotion programme led by migrant women may be one strategy to address the limited participation in preventative healthcare including health screening at the time around menopause.

## Introduction

Australia is a culturally diverse society. The 2021 Australian Census of Population and Housing data showed that nearly a third (29.1%) of the Australian population was born overseas and of these nearly two-thirds have migrated from low- and middle-income countries (Australian Bureau of Statistics, 2021). People leave their country of origins for many reasons. Some voluntarily seek improvement in their own or their family’s circumstances but others are forced to leave due to internal and external conflict, environmental disasters, and risk of persecution (McAuliffe and Triandafyllidou, 2022). In Australia, many women migrants from low- and middle-income countries either enter through the Humanitarian Program for refugees and asylum seekers including the Women at Risk programme or the family and partner visa programme (De Maio *et al.*, 2017). Although migration has many benefits and offers new opportunities, it is also accompanied by losses and unique challenges.

**Table 1.** Demographic characteristics of respondents Data are presented as n (%)

Characteristics	General Practitioner n = 30	Registered Nurse n = 46	Total N = 76^A^
**Sex** ^B^			
Female	19 (63.3)	45 (100)	64 (85.3)
Male	11 (36.7)	0	11 (14.7)
**Age**			
Less than 35 years	1 (3.3)	6 (13.0)	7 (9.2)
35–44 years	11 (36.7)	6 (13.0)	17 (22.4)
45–54 years	10 (33.3)	8 (17.4)	18 (23.7)
55–64 years	7 (23.3)	25 (54.3)	32 (42.1)
65 years and older	1 (3.3)	1 (2.2)	2 (2.6)
**Spoken languages**			
English only	14 (46.7)	29 (63.0)	43 (56.6)
English and another language	16 (53.3)	17 (37.0)	33 (43.4)
**Country of qualification**			
Australia	21 (70.0)	34 (73.9)	55 (72.4)
Other^C^	9 (30.0)	12 (26.1)	21 (27.6)
**Years of clinical experience in Australia**			
≤10	5 (16.7)	10 (21.7)	15 (19.7)
>10	25 (83.3)	36 (78.3)	61 (80.3)
**State of practice**			
New South Wales	6 (20.0)	8 (17.4)	14 (18.4)
Northern Territory	1 (3.3)		1 (1.3)
Queensland	2 (6.7)	2 (4.3)	4 (5.3)
South Australia	2 (6.7)	10 (21.7)	12 (15.8)
Tasmania	3 (10.0)		3 (3.9)
Victoria	12 (40.0)	25 (54.3)	37 (48.7)
Western Australia	4 (13.3)	1 (2.2)	5 (6.6)
**Geographical location of practice**			
Metropolitan	23 (76.7)	30 (65.2)	53 (69.7)
Rural	3 (10.0)	14 (30.4)	17 (22.3)
Remote	4 (13.3)	1 (2.2)	5 (6.6)

A – One participant did not complete the demographic characteristics section

B – One participant did not specify their sex

C – Countries of qualification outside Australia included England, India, Indonesia, Ireland, Nepal, New Zealand, Nigeria, ONG, Philippines, South Africa, Turkey, and the United Kingdom.

Migration to a new country of residence requires becoming familiar with and navigating new systems including healthcare systems. Australia has a two-tiered healthcare system. Medicare is the universal public health insurance scheme which provides free or low-cost subsidised healthcare. In addition, individuals may choose to purchase private health insurance which provides the choice of accessing healthcare outside the public system, but however requires contribution towards the cost of the healthcare.

Primary healthcare in Australia is funded under Medicare and includes health promotion, prevention, early intervention, treatment of acute conditions, and management of chronic conditions and is delivered in diverse settings such as general practices, community health centres, allied health practices, and more recently via communication technologies such as telehealth and video consultations. Primary healthcare is provided by a range of healthcare professionals such as General practitioners (GPs), nurses and midwives, allied health professionals, pharmacists, dentists, and Aboriginal health practitioners.

Health beliefs and behaviours, and expectations of healthcare are shaped by culture. Because of this, migrants’ access to and experiences of healthcare in the destination country are likely to differ from their country of origin which may result in dissatisfaction and disengagement with care (Machado *et al.*, 2022). Additionally, limited health information in community languages, (Robertshaw *et al.*, 2017, Suphanchaimat *et al.*, 2015) inability to communicate proficiently in the destination country’s language and inability to access and navigate the healthcare system (Ahmed *et al.*, 2016) are commonly cited barriers to migrants receiving optimal healthcare.

Health literacy refers to how individuals access and understand information related to health and healthcare, and how they use and act on this information in their daily lives. It is determined by the individual’s skills and capacities as well as the demands and complexity of the healthcare environment. Vulnerable populations such as migrants who speak a language other than that of the host country are more likely to have low health literacy skills which has been shown to result in lower participation in health promotion programmes, preventive healthcare, and higher rates of emergency care and hospitalization (Australian Commission on Safety and Quality in Health Care, 2014).

Stanzel and colleagues (2019, 2020) (Stanzel *et al.*, 2019, Stanzel *et al.*, 2020b) explored menopause-related health literacy and experiences of menopause-related care among women who had migrated from low- and middle-income countries to Melbourne, Australia. They found that migrant women view menopause and associated health changes as a normal life stage and therefore do not see the need to seek healthcare for menopause-related symptoms. Instead, they employ self-care strategies and remedies used in their country of origin to manage any menopause-related symptoms and seek health information mostly from families and friends. In addition, women reported rarely initiating conversations about menopausal health with healthcare providers, because, in some cultures, women’s health, including menopause, is considered an intimate topic not openly spoken about.

It is widely documented that the risks of chronic non-communicable diseases increase in midlife, which in women occurs around the time of menopause (Harlow *et al.*, 2012). Health behaviours such as regular exercise (Anderson and Durstine, 2019) and a balanced diet reduce these risks (Cena and Calder, 2020). Hence, the menopausal transition offers the opportunity to assess and review health and health behaviours to optimize health in older age.

However, in a survey of primary healthcare professionals conducted in 2019, Stanzel et al found that despite most believing that migrant women are interested in health information related to menopause and midlife only about a third routinely offered this to migrant women (Stanzel *et al.*, 2020a). The aim of this study was to gauge primary healthcare providers’ perceptions of barriers and enablers for providing menopause-related care to women who have migrated from low- and middle-income countries.

## Method

### Study design

A cross-sectional study using an anonymous online survey with 17 questions with fixed response options assessed PHCPs beliefs about providing menopause-related care to mirgrant women from low-and middle income countries. Two questions had open-ended response options: (1) ‘Have you identified any additional barriers for discussing menopause-related health with immigrant women?’ and (2) ‘Have you any other suggestions for how comprehensive menopause-related health consultations to immigrant women can be facilitated?’

### Respondents and recruitment procedure

To be eligible to participate in the study respondents had to be a primary healthcare practitioner (PHCP) either a medical practitioner or a nurse working in primary healthcare with experience in providing care to peri- and postmenopausal migrant women from low- or middle-income countries.

Representatives of 15 national- and state-based healthcare professional organizations across Australia, five primary health organizations, and 31 primary health networks were telephoned to promote the survey and distribute it to their members through newsletters and social media.

Those organizations who agreed to distribute the survey were sent a brief introduction and description of the survey, which included a link to the survey and a QR code which could be scanned to access the survey.

Information about the purpose of the study, eligibility criteria, and what participation involved could be accessed via a link. Having read this information, those who were eligible and willing to take part provided implied consent to participate by proceeding to the survey link. The survey was hosted by Qualtrics (Provo, UT, USA) and opened from October 2018 to April 2019. In recognition and appreciation of respondents’ time, those who completed the survey could enter a draw for the chance to receive one of three gift cards each valued at A$100.

### Data source

A study-specific survey with four sections was developed. The questions were informed by findings of their systematic review (Stanzel *et al.*, 2018) and prior research about the health literacy and healthcare needs of migrant women and primary healthcare clinical experience (Stanzel *et al.*, 2019, Stanzel *et al.*, 2020b).

The first section included questions about PHCPs’ perceptions of migrant women’s management of and attitudes towards menopause and menopause-related health literacy; the second asked about PHCPs’ practice relating to the care of migrant women in midlife; and the third investigated PHCPs’ opinions about the barriers and facilitators in engaging with and providing menopause-related healthcare to migrant women, drawing from a pre-specified list. The list of barriers had the following items: lack of confidence working with interpreters; lack of confidence providing care to women from diverse cultural and linguistic backgrounds; lack of culturally and linguistically appropriate resources in community languages; and limited appointment time. These were the items listed as facilitators: onsite nurses with qualifications in women’s health; referral options to women’s health services; a one-stop website which provides health information fact sheets including links to health information websites in community languages; and a Medicare billing number that reflects the time it takes to provide menopause-related care to women from culturally and linguistically background.

In addition, two open-ended questions invited respondents to use their own words to describe what they believed to be barriers or facilitators for providing optimal care. The final section ascertained respondents’ personal and professional characteristics.

The survey was pilot tested by ten PHCPs and researchers and amended based on feedback received.

The results of the quantitative data were published in 2020 (Stanzel *et al.*, 2020a). It reported that most respondents believed that limited appointment time, lack of culturally and linguistically appropriate menopause information, and lack of confidence about providing menopause care were cited as barriers to proving menopause care. To address these, a one-stop website with health information in community languages, a specific Medicare billing number that acknowledges the length of time it takes to provide menopause-related care to migrant women and referral options to practitioners with qualifications and expertise in women’s health were suggested.

### Data management and analysis

Responses to the free-text questions were collated in a Word document and analysed using thematic analysis, as described by Braun and Clarke (2006). This method involves familiarization with data through transcription and repeated readings; assignment of initial codes based on aspects related to the research topic; arrangement of codes into preliminary themes based on broader patterns; refinement of preliminary themes into final themes; and finally production of the report.

A manual, inductive coding process was used to generate codes, which were then grouped into preliminary themes. These were then refined further into final themes, which were arranged to form a cohesive narrative. Codes and themes were discussed within the research team at each stage until consensus was reached. Once the thematic structure was finalized, representative quotations were selected to reflect the thematic interpretation.

### Ethics

The study was approved by the Monash University Human Research Ethics Committee (MUHREC 15095).

## Results

Of the 245 respondents who clicked on the survey link, 106 discontinued after reading the participant information which described that only PHPC were eligible if they provided menopause-related care to women who had migrated from low- and middle-income countries. Of the remaining 139 respondents, 117 completed the entire survey, of which 76 (65%) answered either one or both open-ended response questions. Of these, 30 were general practitioners (GPs) and 46 registered nurses (RNs). More than half of GPs spoke a language other than English, most had completed their training and obtained their qualification in Australia and had practised for more than 10 years. Nurses practised in a range of roles including Sexual and Reproductive Health Nurse (SRHN), Community Health Nurse (CHN), Women’s Health Nurse (WHN), Practice Nurse (PN), Nurse Practitioners (NP) and Refugee Health Nurse (RHN), most had obtained their qualification in Australia and just over half was 55 years or older (see Table 1).

Three themes were identified: Migration experiences, Healthcare environment, and Culture shape health beliefs and behaviours.

### Migration experiences

Competing priorities were identified by many as a barrier to migrant women accessing menopause-related care. Respondents noted that the migration experience and its associated loss of social networks and family support coupled with the additional responsibilities associated with settlement makes seeking healthcare a low priority. The women prioritize their family’s needs over their own self-care.

*‘Many immigrant women face difficulties of settlement/health/finances/languages/etc, which outweigh hot flushes and sleepless nights – they prioritise their needs and menopause is often low on the list’ (GP, Female, 45-54)*

*‘Women at this time of life are often too busy taking care of their families to remember to make that appointment from the brochure still sitting in her purse’ (RN, Female, <35)*

When migrant women do make time to seek healthcare, it is for more complex health issues.

*‘Other co-morbidities take priority – menopause discussion is often pushed down the priority list’ (GP, Male, 35-44)*

Respondents identified the need for innovative and flexible approaches to healthcare and health promotion to address these barriers. Consistent follow-up from previous appointments, outreach clinics, attending community groups to provide health promotion programmes, and offering free transport and childcare on the premises were some of the suggested solutions.

*‘Sometimes I think we need to be more flexible in how we provide information e.g. we need to go where women meet and offer services there e.g. clinics at ESL [English as second language] schools or attending women’s groups. We can provide information and assist women to arrange appts’ (CHN, Female, 55-64)*

*‘Need for childcare availability to enable clients who are caring for grandchildren’ (SRHN, Female, 55-64)*

### Healthcare environment – the health system’s capacity to provide culturally competent care

Respondents commented on the unique barriers migrant women encounter when accessing health services, in particular for women for whom English is not a first language.

*‘In my workplace, arranging appointments can be a barrier. Women who don’t speak English are expected to call for appointments. There is no ability for them to walk in [to the service] to make an appointment’*

*(CHN, Female, 55-64)*

In addition, time-constrained consultations were identified in limiting the ability to provide additional health-promoting information.

*‘There is often not enough time to discuss preventive health or explore issues that are outside of their presentation such as menopausal symptoms which many women see and accept as part of life’ (GP, Female, <35)*

To overcome this, respondents suggested arranging follow-up menopause-specific consultations if concerns were raised during shorter appointments. This allows practitioners to prepare for these longer consultations and provide more comprehensive care.

*‘If a woman sees a GP for any reason, and the doctor recognises symptoms of menopause, there should be an option for an immediate booking of either a longer appointment if the woman is comfortable speaking with that clinician or an appointment with a women’s health service of the client’s choice’ (RN, Female, <35)*

Limited health promotional resources for culturally and linguistically diverse people were another barrier commonly noted by respondents.

*‘Language specific menopause information is minimal’ (SRHN, Female, 55-64)*

Thus, respondents identified how health promotional materials in diverse community languages would facilitate the provision of menopause-related care to migrant women.

*‘Access to in-language health information would assist in relaying information to the patient. Using labelled anatomical pictures in English and the preferred language. A glossary of medical terms e.g. cervical screen/cervix/uterus in different languages…’ (WHN, Female, 55-64)*

Although written resources were considered helpful, limited literacy skills and IT competency were identified as additional barriers.

*‘Most of the women settled here have had little or no education and [are] illiterate in their own language, therefore written resources are not useful. They do not have access to computers, nor the skills to use [them]’ (RHN, Female, 55-64)*

Respondents emphasized the need for innovative health promotional strategies, and some of the suggested strategies included health-related advertisements on television and radio, and group education sessions.

*‘A series of health-related advertisements in the general community, such as waiting room tvs, radio, regular tv, internet, newspapers and magazines’ (SRHN, Female, 45-54)*

Recognising their limited training and skills in providing culturally competent care made the provision of care to migrants difficult.

*‘There needs to be cross cultural (sensitivity and responsive) service delivery training to all service providers (including doctors). Service providers are equally responsible for the delivery/establishment of a culturally responsive service system’ (CHN, Female, 55-64)*

Some respondents recognized their limited resources and skills and identified women’s health services, nurse practitioners, and women’s health nurses as referral options to provide migrant women with optimal menopause-related care.

*‘Within a specialist women’s health setting which provides the full range of women’s health services such as contraception, … as well as menopause management’ (GP, Female, 55-64)*

*‘Nurse Practitioners in women’s health and Women’s Health nurses are ideal’ (Female, 55-64, NP)*

### Culture shapes health beliefs and behaviours – including health-seeking behaviours

In some cultures, menopause and midlife are considered normal and a welcomed life stage, signalling the end of the reproductive role, the inconveniences of menstruation, and the beginning of greater social status as a respected elder within their community. Respondents in this study made similar observations and commented that this belief may act as a barrier to seeking healthcare for symptoms related to midlife health.

*‘In my experience, many immigrant women see menopause as a normal part of life, not a medical condition, so [it is] not worth mentioning to the doctor’ (GP, Female, 45-54)*

*‘Not to generalise, but often immigrant women are used to putting up with more discomfort than Australian born women. This acceptance of suffering often leads them to ignore menopause symptoms as simply a part of life’ (RN, Female, <35)*

The conversation about women’s health including physiological function of the body, sexual and reproductive health, and menopause may be unacceptable or as one respondent alleged, even illegal in some countries as observed:

*‘It is taboo in some societies to speak about woman problems’*

*(Mental Health Nurse, Female, 55-64)*

*‘Hazara women have had no information about their bodies except from direct experience; I am told it is illegal in Afghanistan to teach a woman about her body function’ (GP, Female, 55-64)*

PHCP’s sex was cited as an additional barrier. Male providers commented on migrant women either requesting to see a female provider or apologising for seeing a male provider for a women’s health-related issue.

*‘I sometimes found that women even “apologise” to see me (a male doctor) and ask for a medical certificate due to severe period pain – an issue they only want to discuss with female doctors. What we (healthcare providers) are very comfortable to discuss may be a very embarrass[ing] issue to migrant women’ (GP, Male, 55-64)*

A reluctance to speak openly about their health in front of men including male family members accompanying women for their appointments was observed by respondents.

*‘These women are often difficult to assess as often they are with husbands and will not discuss [it] in front of them or will not discuss with male nurse or doctor’ (RN, Female, 55-64)*

The Global Expenditure on Health Report published by the World Health Organization (2021) found that although primary healthcare spending accounts for about half of government health spending in low- and middle-income countries most primary healthcare spending went to outpatient curative care and medical goods. Only a small proportion is allocated to preventive care (World Health Organization, 2021). Conversely, in Australia early detection and prevention of diseases is a prominent feature of primary healthcare (Department of Health and Aged Care, 2023). Respondents observed a dissonance between migrant women and PHCPs’ expectations of healthcare delivery. Whilst migrant women mostly sought care for symptoms of illnesses, providers wanted to deliver more holistic care. Respondents believed that migrant women become suspicious of PHCPs who attempt to engage them in conversations about health promotion or preventive health.

*‘Some of these women are also apprehensive about doctors as they sometimes find questions about their health outside of their presenting complaint as potentially intrusive or question the relevance to their presentation, or worry that the doctor will ‘find something wrong with me’ and can sometimes be reluctant to come to doctors’*

*(GP, Female, <35)*

Migrant women often present when illness-related symptoms are severe, making care more complex and time consuming. Consequently, PHCPs prioritize managing these health issues before commencing a conversation about health-promoting behaviour.

*‘Immigrant women tend to come to doctors when they are quite symptomatic with a medical issue that is often more complex than anticipated’ (GP, Female, <35)*

Respondents mentioned how in some migrant communities, Western medicine, including pharmaceuticals, is believed to be harmful and that this leads to reluctance to take prescribed medications and seeking medical care.

*‘They are culturally less likely to be accepting of hormonal intervention for fertility control let alone menopause. No point mentioning it to the doctor if you don’t want medicine’ (GP, Female, 45-54)*

*‘A lot feel western medicine is taboo/harmful, communities and cultures discourage women from receiving treatment’*

*(SRHN, Female, <35)*

Respondents thought that rather than seeking medical advice, migrant women are more likely to trust women from their communities and rely on information from female peers and family members. This was perceived as problematic as information from family and friends may not be evidence-based and might promote harmful practices.

*‘They talk to their friends [and] neighbours who are as much in the dark about reliable information as they are’ (GP, Male, 55-64)*

To promote evidence-based information, small group sessions within their community facilitated by female practitioners or female members from their community and delivered in community languages were identified as a culturally competent health promotion strategy to facilitate menopause-related care for migrant women.

*‘Perhaps having a member from their community/language group who can give health information education sessions about menopause (i.e. like a health worker) may remove culturally sensitive barriers and help open up conversations prior to seeing a nurse or doctor’ (GP, Female, 35-44)*

*‘Local discussion groups/information sessions offered on women’s health/menopause in local immigrant women’s languages, with bilingual health educators, and/or interpreters’ (SRHN, Female, >65)*

Given the diverse experiences and needs of migrant women, respondents stressed the need to collaborate with community groups when developing culturally appropriate health promotion strategies and involving migrant women in its delivery.

*‘Liaising with the target immigrant populations women’s networks (i.e. via religious groups) for input and guidance’ (PN, Female, 45-54)*

*‘Involve and train women from these backgrounds to deliver the messages in a culturally safe space that not just informs the women but also empowers the women’ (CHN, Female, 55-64)*

## Discussion

This study identified barriers and enablers of health promotion and preventive care for migrant women in midlife from the perspective of primary healthcare providers. The finding that PHCPs attributed barriers to the migration experience, the healthcare environment, and the culture in women’s country of origin indicates that a multipronged approach is needed to improve healthcare and the future health of women from culturally and linguistically diverse backgrounds.

The migration experience varies greatly among migrants depending on the reason for migrating and the circumstances under which migration took place. PHCPs identified priorities related to settlement and family reasons why migrant women are less likely than other women to engage in preventive healthcare. For financial reasons, migrant women may need to enter the paid workforce for the first time. In addition, they are likely to continue to be responsible for unpaid work related to their traditional gender roles including providing care to family members and domestic duties (Rezazadeh and Hoover, 2018). Anderson (1987) (Anderson, 1987) refers to the ‘double burden’ where migrant women feel overwhelmed by the additional workload and the absence of extended family and community support often resulting in lack of self-care. This may be one of the reasons why migrant women report less participation in preventive healthcare than their native-born counterparts. (United Nations Development Programme, 2018)

In Australia, primary healthcare is largely provided by GPs. The current primary healthcare funding model favours short consultations which according to study respondents is insufficient for the time that is required to discuss health-promoting strategies with migrant women. They suggested that a model of care in which allied health practitioners offer health promotion consultations to women might be one way in which health promotion in primary care setting could be improved. Another model of care to address workforce shortages is the process of ‘task-shifting’ which has been introduced in healthcare (Joshi *et al.*, 2014). Task-shifting refers to the process of transferring responsibility of a task usually performed by one professional to another with different educational and training background or to people trained to specifically perform the task within a limited scope of practice. (Joshi *et al.*, 2014, van Schalkwyk *et al.*, 2020). This process has been shown to provide benefits but also challenges to the health system. In a review of systematic reviews by Leong et al (2021) (Leong *et al.*, 2021), the authors concluded that task-shifting from doctors to nurses improved access to care including health promotion and health education.

In addition, the review found that task-shifting to nurses led to better outcomes in the management of non-communicable chronic conditions, higher return rates for follow-up appointments, and is viewed favourably by patients living with chronic diseases. However, for interventions that were considered as more ‘medical’ or ‘complex’, patients preferred to be treated by a doctor. Task-shifting from doctors to nurses for minor illnesses was reported to be cost-effective, and it allowed doctors to attend more complex cases which most likely would result in staff cost saving and consequently benefit the healthcare system. Although task-shifting is generally considered cost-effective, the lack of clarity on the financial roles and responsibilities of the national and regional health authorities acted as a barrier.

Limited training and lack of confidence in providing culturally competent menopause-related care were reported by some respondents. To address this, they recommended referral options to specialist women’s health practitioners. Rather than transferring care to a specialist service, the Interprofessional Collaboration (IPC) model of care may provide an alternative approach to care. IPC involves integrated cooperation and collaboration among various health professionals. It is becoming more common in primary healthcare due to the increasingly complex needs of patients. Although a systematic review about the effectiveness of IPC in primary care has shown mixed results, the authors concluded that IPC in primary healthcare can be effective, but further investigation is needed (Carron *et al.*, 2021). The barriers and facilitators of IPC were examined in a review by Rawlinson and colleagues (2021) (Rawlinson *et al.*, 2021). The authors noted that funding, lack of political will, insufficient legal support for role expansion and limited human resources to implement and oversee this model of care were the most frequently cited barriers.

Facilitators for IPC in this review were effective and clear communication among healthcare professionals, teamwork with like-minded colleagues, and a supportive organization that fosters interprofessional collaboration and development.

Respondents noted that migrants were reluctant to accept Western medicine and were more likely to turn to community members for health-related information. Training migrant women to deliver health information to other women in their communities is a culturally and linguistically appropriate health promotional strategy which has shown success in other areas (Ponce-Gonzalez *et al.*, 2021). Respondents suggested that this could be an enabling strategy for also delivering health-promoting messages to migrant women in midlife.

Culture shapes perceptions of healthcare. In low- and middle-income countries, primary healthcare spending largely focuses on curative care and medical goods and less on preventive care. Hence, the concepts of health promotion and prevention of non-communicable conditions may be unfamiliar to migrants from such countries. Study respondents observed that women only sought care for acute illness or when symptoms were severe. Mengesha et al (2017) (Mengesha *et al.*, 2017) who investigated healthcare practitioners’ perceptions of providing sexual and reproductive care to migrant women made similar findings. They reported that women mainly accessed care for illness and were unfamiliar with health promotion and health screening. Yet, midlife is an ideal time to assess and promote positive health behaviours, including attending health screening, to optimize health in older age. Unfamiliarity with preventive healthcare may mean that migrant women miss out on opportunities to maintain and improve their health.

## Strengths and limitations

The study has several strengths. The survey was distributed widely and made accessible to all Australian PHCPs who are members of relevant professional associations and networks. As a result, the experiences and perspectives of PHCP from across Australia, working in diverse settings, with a range of different characteristics were captured. A high proportion of respondents volunteered their comments which varied in depth and lengths from brief one-sentence comments to more detailed descriptions about the barriers and facilitators of menopause-related healthcare. In addition, Krippendorff (2004) (Krippendorff, 2004) proposes that one of the best ways to judge the quality of the findings is by evaluating whether novel insights into the studied experience have be provided and if so whether this will increase the understanding of the phenomena and inform practice. Our study provides unique understanding of how menopause-related care could be improved if the barriers as identified by PHCP could be addressed.

Nevertheless, we recognize the following limitation. Qualitative research is concerned with investigating social phenomena and the meaning people attribute to their experiences. There are a number of strategies to assure quality in qualitative research (Hammarberg *et al.*, 2016). To validate the meaning of responses, investigators might paraphrase the reply or ask explicatory question. However, written responses do not allow investigators to ask follow-up questions to ensure the meaning of responses is fully understood. In order to support the credibility of this study, the data analysis was completed by two investigators independently. Findings were reviewed and discussed until agreement was reached.

## Implications for policy, practice and research

Understanding the barriers and facilitators to providing menopause-related care to migrant women can inform strategies to improve migrant women’s access to culturally sensitive care that meets their needs.

The Australian National Preventive Health Strategy 2021-2023 recognized that people from culturally and linguistically diverse backgrounds experience a higher burden of disease than their native-born counterpart. This is not due to personal choices but rather to circumstances including social disadvantage. Preventive healthcare needs to be flexible and allow for sufficient time to cater for the additional needs of people with social and economic disadvantages including those from culturally and linguistically diverse backgrounds. A review of the current primary care funding model is needed to address the shortcomings of the existing model.

Community groups, education, employment, and support services for migrants provide important opportunities to connect with migrant communities. Exploring ways to collaborate with these local groups and services to implement or offer health promotion programmes in women’s native languages may improve access to valuable health information and resources.

Migrants who enter Australia on a humanitarian visa have access to settlement workers who support newly arrived migrants to learn and become familiar with Australian systems, including the healthcare system. Yet, for migrants who enter on a family or partner visa this service is not available and they rely on their partner or family member to help them navigate systems. Further research is needed to explore the information and support needs of women who enter Australia on a partner visa to inform strategies to meet these needs.
